# Evaluating the implementation of the perinatal maternal route in a group of students of the psychology program of two Universites in Colombia during the period 2022

**DOI:** 10.1192/j.eurpsy.2023.1903

**Published:** 2023-07-19

**Authors:** E. P. Ruiz Gonzalez, M. N. Muñoz Argel, J. J. Vicuña Romero, T. Noguera Morales, M. Y. Acevedo Rodríguez

**Affiliations:** 1 Universidad Pontifica Bolivariana, Montería; 2 Universidad Pontifica Bolivariana, Medellin; 3 Universidad Pontifica Bolivariana, Palmira; 4 Universidad Pontifica Bolivariana, Bucaramanga, Colombia

## Abstract

**Introduction:**

Reducing maternal and newborn mortality is apriority on the health agenda. Priority, integral, integrated and barrier-free care a attention to the population frames the spirit of the route. The Comprehensive Care Route in Perinatal Maternal Health (CRPMH) proposes “to promote health and the improvement of maternal and perinatal health outcomes, through comprehensive health care, including coordinated action FROM the state, THE society and the family on the social and environmental determinants of health inequities” (Minsalud).

**Objectives:**

To evaluate the implementation of the CRPMH in a group of maternal students from 0-12 months.

**Methods:**

Qualitative, through semi-structured interview techniques and focused groups (FG) referenced from the CRPMH (table 1) in 11 undergraduate students in psychology over 18 years from 2 Colombian universities.

**Results:**

Qualitative analysis evaluates convergences/divergences by percentages of questionnaire responses and axial text analysis (FG). In preconception attention 100% of mothers do not report signs of health risk, however, caesarean section was performed in 83.3% of cases, this safer method is perceived for the mother and fetus, and is justified taking into account that the pain of childbirth is very strong (FG).

In gestational health they indicate prenatal control, medical appointments, formation in the condition of the fetus, guidelines on care, respectful upbringing and breastfeeding in 100%. In contrast, the focus group reports low empathy of doctors toward levels their fears, reduced time to address concerns, negative information about labor and satisfaction with medical procedures, considering caesarean section a humanized strategy.

Access to CRPMH is known by 50% of mothers, they do not know the preconceptional consultation. In the GF they conclude that the information on preparation for maternity and paternity is ineffective.

Psychological support is absent during childbirth and postpartum. There is a greater knowledge about breastfeeding 83.3%

**Discussion:**

The successful implementation of the route could reduce the risks of physical and psychological impact on perinatal maternal health by facilitating decisions about motherhood and its practice in the university educational environment. There was recognition of the clinical factors of the CRPMH and ignorance of the responsibility of the educational environment in its implementation.

**Image:**

**Figure:**
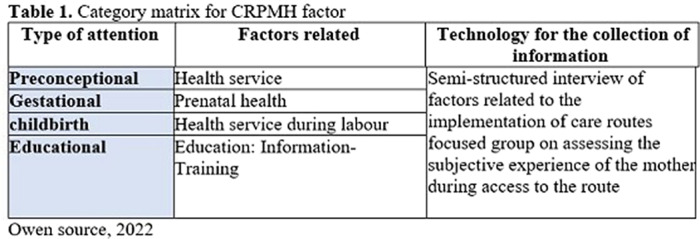

**Conclusions:**

The CCRPMH regulation is insufficient to guarantee its implementation. Risk factors include the quality of service provided by health-care providers and the lack of knowledge of regulations in university management.

**Disclosure of Interest:**

None Declared

